# Chasing a Breath of Fresh Air in Cystic Fibrosis (CF): Therapeutic Potential of Selective HDAC6 Inhibitors to Tackle Multiple Pathways in CF Pathophysiology

**DOI:** 10.1021/acs.jmedchem.1c02067

**Published:** 2022-02-11

**Authors:** Simona Barone, Emilia Cassese, Antonella Ilenia Alfano, Margherita Brindisi, Vincenzo Summa

**Affiliations:** Department of Pharmacy, Department of Excellence 2018-2022, School of Medicine and Surgery, University of Naples “Federico II”, Via D. Montesano 49, I-80131 Naples, Italy

## Abstract

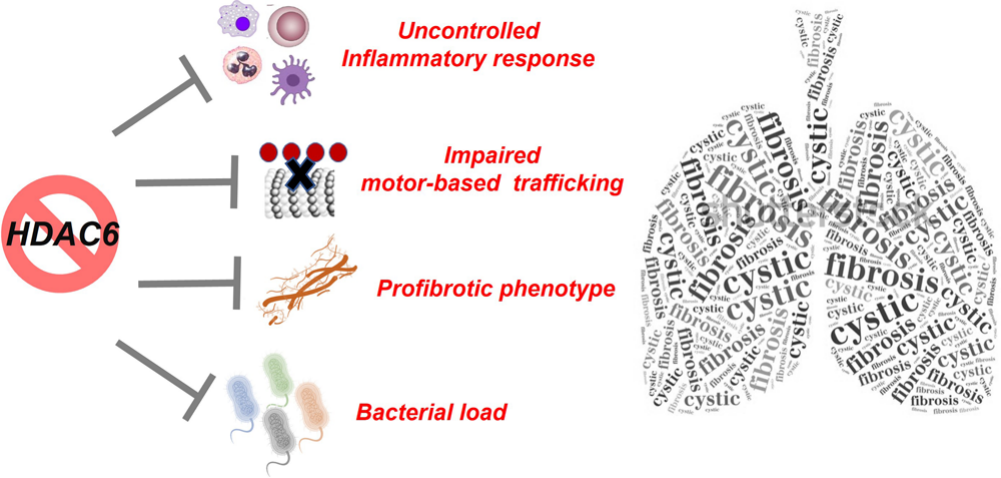

Compelling new support has been provided for histone deacetylase isoform 6 (HDAC6) as a common thread in the generation of the dysregulated proinflammatory and fibrotic phenotype in cystic fibrosis (CF). HDAC6 also plays a crucial role in bacterial clearance or killing as a direct consequence of its effects on CF immune responses. Inhibiting HDAC6 functions thus eventually represents an innovative and effective strategy to tackle multiple aspects of CF-associated lung disease. In this Perspective, we not only showcase the latest evidence linking HDAC(6) activity and expression with CF phenotype but also track the new dawn of HDAC(6) modulators in CF and explore potentialities and future perspectives in the field.

## Introduction

Cystic fibrosis (CF) patients present mutations of the cystic fibrosis transmembrane conductance regulator (CFTR) gene, which codes for a cyclic AMP-regulated chloride ion channel.^1^ Loss of function of this channel leads to defective transport of chloride ion across the surface of epithelial cells, which in turn interferes with the clearance of inhaled microorganisms. This lack of defense against microorganisms is responsible for severe airway infections in CF patients.^2^ Intensive antibiotic therapy is mandatory for maintaining lung function and quality of life and has contributed to a significant extension of the mean expected lifetime of CF patients.^3^ The CF-associated complex microbial flora of the respiratory tract are characterized by the coexistence of multiple bacterial species, which complicates the identification of a very efficacious antibacterial treatment. Infections triggered by *Pseudomonas aeruginosa* (*PA*) are particularly challenging and have the tendency to persist and become chronic.^4^ Morbimortality of CF patients is largely ascribable to chronic lung infection, inflammation, and uncontrolled fibrotic tissue rearrangement. The underlying pathways still remain underexplored; additionally, the efficacy of currently employed anti-inflammatory treatments is limited to symptomatic management of CF airway inflammation.^5^

The initial stage of CF-related lung disease mainly consists of prolonged airway inflammation accompanied by protracted infections, chronic inflammation, mucus hypersecretion, and oxidative stress.^6−8^ Evolution to chronic CF disease entails recurrent or steady infection with *PA*, which finally results in chronic airway inflammation, permanent pulmonary impairment, and collapse of respiratory function.^9^ CFTR malfunctioning is also related to a defective autophagy process that promotes chronic inflammation and oxidative stress in CF airways.^10,11^ Chronic inflammation is mainly associated with NFκB-mediated proinflammatory signaling (as demonstrated by increased levels of the NFκB-dependent genes KC and Mip-2) and IL-8-dependent neutrophil chemotaxis.^6,8^ Beyond neutrophils, other innate and adaptive immune cells are linked to increased levels of pro-inflammatory cytokines and chemokines, thus promoting CF-related lung disease;^12^ therefore, a deeper knowledge of the mechanisms underlying inflammatory response is of pivotal importance in CF research. Since CFTR-targeted therapies display swinging efficacy in reducing inflammation in CF airways, a complementary anti-inflammatory therapy to be employed in association with CFTR correctors and potentiators would be an added value in this context. More importantly, patients harboring CFTR mutations that are not responsive to currently available modulators would at least take advantage of innovative therapeutic options to re-establish inflammatory regulation.

It has also been demonstrated that CF fibroblasts exhibit an aberrant phenotype characterized by uncontrolled proliferation and myofibroblast differentiation, increased sensitivity to growth factors, and altered levels of proinflammatory and fibrotic mediators. In particular, CF lungs show increased release of transforming growth factor-β1 (TGF-β1), a multifunctional protein implicated in wound repair, epithelial-to-mesenchymal transition (EMT), myofibroblast differentiation, and production of various elements of connective tissue matrix.^5,13^

The latest studies identified the histone deacetylase (HDAC) class of enzymes as strategic components of the complex molecular machinery underlying both inflammation and fibrogenesis in CF. Accordingly, compelling new support has been provided for isoform HDAC6 as a common thread in the generation of the dysregulated proinflammatory and fibrotic phenotype in CF.^14−19^ HDAC6 also plays a crucial role in bacterial clearance or killing as a direct consequence of its effects on CF immune responses.^18^ Inhibiting HDAC6 functions thus eventually represents a novel and effective strategy to tackle multiple aspects of CF-associated lung disease. Selective HDAC6 inhibition should also avoid the common toxicities related to the currently available unselective HDAC inhibitors.

## 1 Current Therapeutic Strategies in Cystic Fibrosis: State of the Art and Unmet Needs

CF is characterized by a complex clinical picture. To date there is no cure for CF, and the current treatment regimen requires ad personam approaches. In general, a multidrug combination is required to control the symptomatology associated with this pathology and to ensure improved quality of life and life expectancy in CF patients.^20^

Currently, CF treatment includes CFTR modulators, antibiotics, bronchodilators, mucolytics, and anti-inflammatory and food supplements (because of the lack of pancreatic enzymes and vitamins in CF patients).^21^

CF patients also deal with chronic bacterial infections. Pulmonary infections are especially caused by *PA* and *Staphylococcus aureus* (*SA*). Even if primary infection is characterized by a low bacterial load, these types of infections easily become chronic because of reduced mucociliary clearance (caused by CFTR alteration), superficial airway fluid hypertonicity, and resistance toward β-lactam antibiotics, mainly due to *PA* strains that produce serine and metal β-lactamases.^22,23^ It is also important to underline how chronic infection in turn prompts pulmonary tissue destruction, thus further worsening the clinical picture for CF patients. Therefore, the role of antibiotic therapy is crucial to control *PA* and other CF-associated bacterial strains.^24,25^

**Figure 1:**
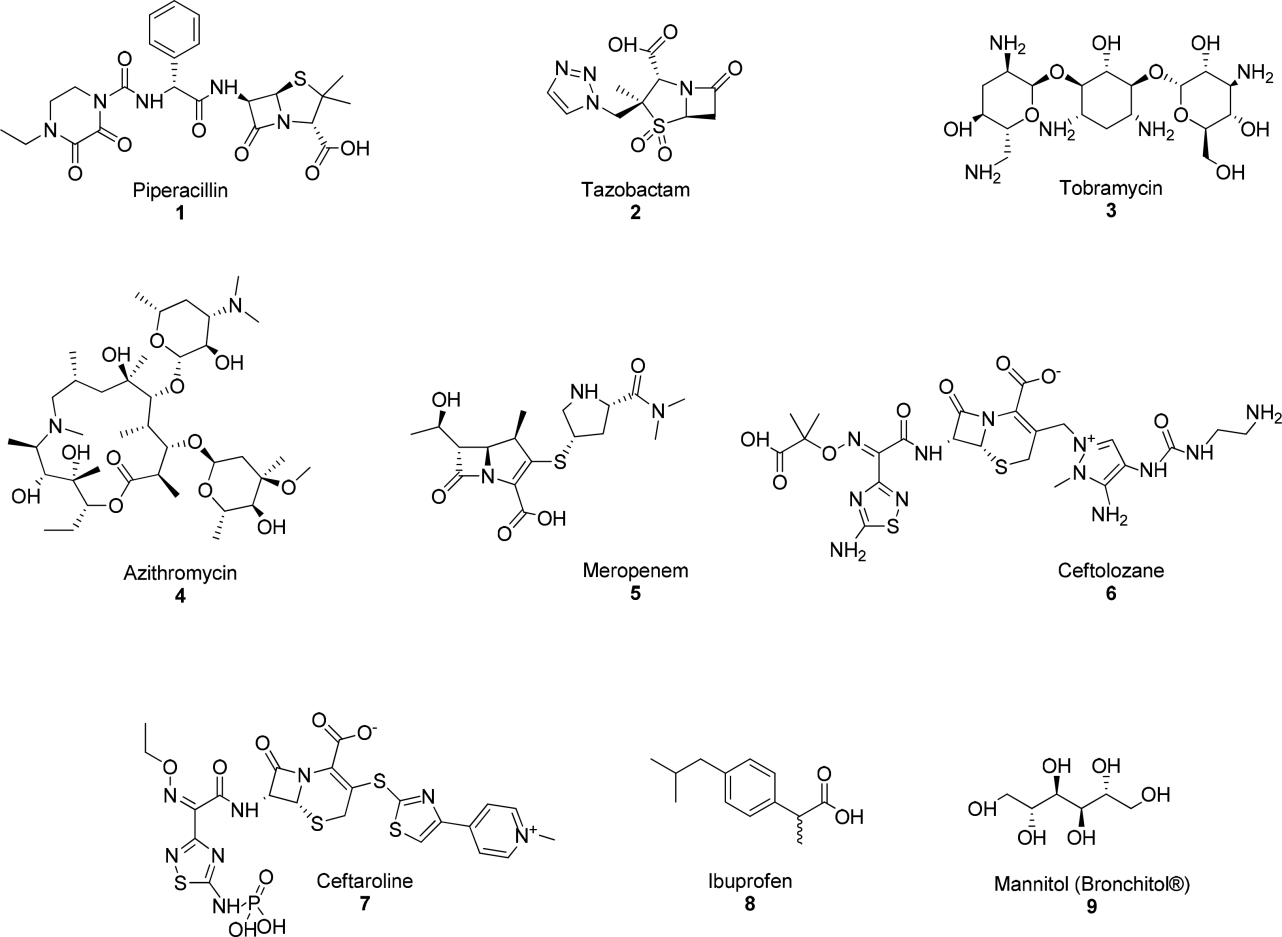
Chemical structures of compounds **1**–**9** used as antibiotic and anti-inflammatory drugs in CF therapy.

*PA* infections in CF are generally treated with β-lactam antibiotics. Piperacillin (**1**, Figure 1) is among the more widely used, and it is often associated with tazobactam (**2**, Figure 1), aminoglycosides such as tobramycin (**3**, Figure 1), macrolides such as azithromycin (**4**, Figure 1), and carbapenems such as meropenem (**5**, Figure 1). Tazobactam shows efficacy in the inhibition of serine β-lactamases produced by *PA*.

Fifth-generation cephalosporins such as ceftolozane (**6**, Figure 1), associated with tazobactam, show good activity against *PA* and variable activity against other Gram-negative bacteria;^26^ ceftaroline (**7**, Figure 1) is effective against methicillin-resistant *SA* (MRSA).^27^

A more difficult therapeutic approach involves bacterial strains expressing metallo β-lactamases, since no drugs are currently approved for their specific inhibition.

In addition to its effect on bacterial protein synthesis, azithromycin has important immunomodulatory and anti-inflammatory properties, which in part could explain the effectiveness of this class of antibiotics. In fact, in patients with persistent *PA* infection, azithromycin strongly reduces neutrophil chemiotaxis in the lung and the release of elastases by neutrophils themselves.^28^

Another important aspect related to the management of microbial infections in CF patients is related to nontuberculous mycobacteria (NTM) present in roughly 10% of CF patients, although only a small set of them will show NTM disease. However, patients potentially prone to develop NTM lung disease will just need careful monitoring on a precautionary basis if symptoms and radiographic outcomes are negligible.^29^

With regard to anti-inflammatory therapy, ibuprofen (**8**, Figure 1) is certainly the most widely used drug, and it is able to reduce the migration of neutrophils to the lung.^30^ However, its use is limited because of side effects (e.g., renal failure, gastric ulcers), which are common to all nonsteroidal anti-inflammatory drugs (NSAIDs) and are associated with the chronic intake required for CF-related inflammation treatment. Moreover, the need to monitor the blood concentration to ensure the therapeutic effect for long times is an additional deterrent to the use of ibuprofen. Another important factor to be considered when dealing with both steroidal anti-inflammatory drugs and NSAIDs is the interindividual variability, which significantly affects treatment efficacy and tolerability.

CF patients also experience the presence of thick, sticky mucus. For this reason, mucolytic therapy is considered to be beneficial to reach the lower respiratory tract, where thicker mucus is produced. Bronchitol (**9**, Figure 1) is the most representative and used drug of this category.

**Figure 2:**
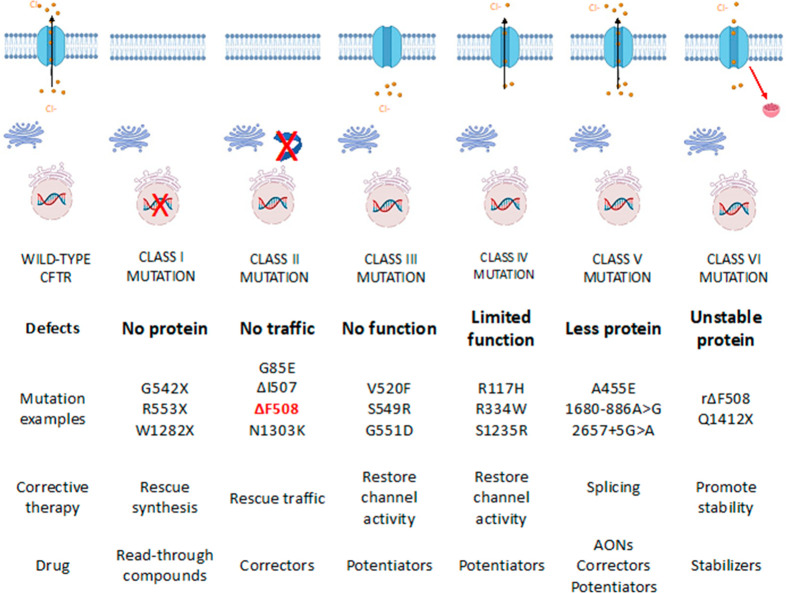
Summary of CFTR mutations and related pharmacological therapy. AONs = antisense oligonucleotides.

The development of CFTR modulators marked the most important breakthrough in CF therapy. Depending on the mechanism of action, they are classified as enhancers, correctors, or amplifiers. Correctors and enhancers act on mutations that involve a defect in CFTR maturation and migration on the cell membrane. Amplifiers act to increase CFTR protein production, regardless of the type of mutation; a greater amount of defective protein available would make the action of correctors and enhancers more profitable (Figure 2).^31^

**Figure 3:**
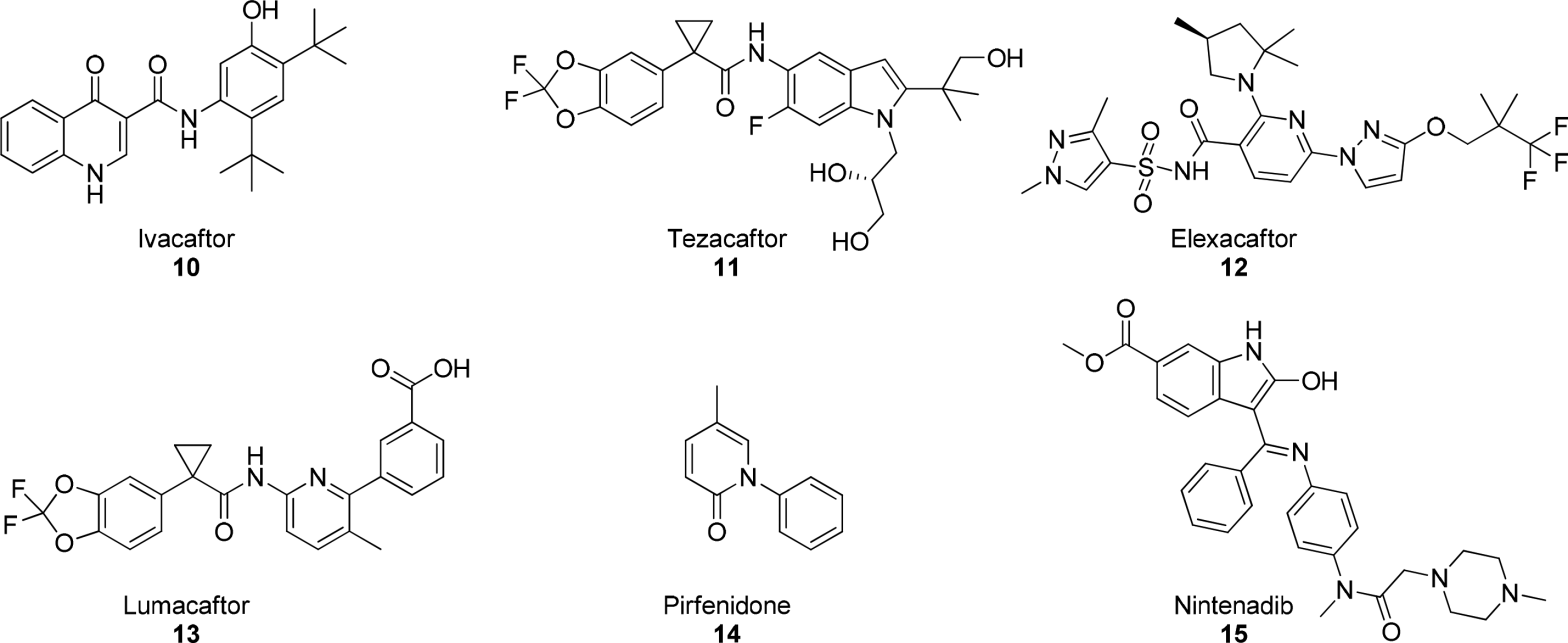
CFTR modulators (compounds **10**–**13**) and antifibrotic drugs (compounds **14** and **15**) used in CF therapy.

Symkevi is a combination of ivacaftor (**10**, Figure 3) and tezacaftor (**11**, Figure 3) and is approved for patients (>12 years old) who are homozygous for phenylalanine 508 deletion (ΔF508). This mutation is associated with a defect of CFTR transport from the nucleus to the cell membrane and with reduced stability of the channel in the cell membrane itself. CFTR correctors are basically able to increase the cell-surface expression of mutated CFTR. Ivacaftor is an effective enhancer for class III CFTR mutations (channel opening defect); it acts by improving CFTR channel opening, thus increasing chlorine transport and leading to cell osmolarity within physiological parameters.

Tezacaftor is a CFTR corrector and improves CFTR protein folding and transport to the cell membrane. Thus, it is useful for patients bearing mutations that result defective in protein folding. Kaftrio, the last treatment approved, is a combination of ivacaftor, tezacaftor, and elexacaftor (**12**, Figure 3) and acts as corrector of CFTR defects, while Orkambi combines lumacaftor (**13**, VX809; Figure 3) and ivacaftor and is used in pediatric patients that are at least 2 years of age. CFTR modulators are essential drugs in CF therapy, but because of the large number of CFTR mutations, they unfortunately are not always effective in the entire patient population. Furthermore, their use is restricted to patients harboring the specific mutations for which their use has been approved.

Pirfenidone (**14**, Figure 3) and nintedanib (**15**, Figure 3) are used in the therapy for idiopathic pulmonary fibrosis (IPF) and represent another relevant therapeutic option to be considered in CF, with respect to CF-associated pulmonary fibrosis.^32^ In IPF these drugs showed efficacy in reducing fibrotic progress and pulmonary failure, delaying disease progression without reversing lung damage. However, their mechanism of action is still unclear, and therefore, more detailed studies are required for their safe and effective use in CF treatment.^33^

Ideally, an effective drug for CF should act independently of interindividual gene variability and should be suitable for chronic therapy and therefore characterized by a wide therapeutic window and high tolerability by the patient. Currently, none of the available drugs used in CF therapy possess all of these characteristics. Therefore, further research efforts are still necessary to identify more effective and safe therapeutic options in CF.

## 2 Epigenetic Regulation in Cystic Fibrosis

Epigenetic regulation is a complex mechanism involved in gene expression changes due to alterations of chromatin packaging. The main epigenetic mechanisms include histone modification and DNA methylation, among others.^34,35^ Specifically, acetylation, methylation, phosphorylation, and ubiquitination are the most representative histone modifications, and they are implicated in several physiopathological processes. Pairs of enzymes with opposing activity are responsible for fine-tuning these modifications, such as histone methyltransferases (HMTs) and demethylases or histone acetyltransferases (HAT) and HDACs.

These dynamic and reversible modifications regulate cell-specific gene expression, acting as promoters or repressors. In particular, histone acetylation causes gene activation, while histone methylation can silence or activate genes.^36,37^

To date, the role of epigenetics in CF has not yet been entirely unveiled, although several studies have pointed out the relevance of DNA methylation and histone modifications in CFTR gene regulation and protein activity.^38,39^ These mechanisms could be crucial for the discovery of innovative therapeutic strategies in CF treatment.

A study highlighted the relationship between CFTR mutations and microRNA (miRNA)-controlled pathways.^35^ miRNAs are single-stranded noncoding RNAs that regulate cell differentiation, growth, and proliferation as well as apoptosis and gene expression.^40^ CFTR plays a pivotal role in cellular homeostasis by regulating Cl^–^ and HCO_3_^–^ ingress and egress, and the concentrations of these ions influence miRNA expression, causing abnormal lung epithelial remodeling and altered immune response in CF patients. Altered miRNA pathways could explain major disorders associated with CFTR mutations.^35^ Thus, miRNAs could be a novel therapeutic target for the management of CF and CF-related pathologies such as diabetes, liver fibrosis, and pancreatic adenocarcinoma.

DNA methylation and histone deacetylation promote CFTR transcriptional inhibition. DNA methylation at promoters represses gene expression, and this mechanism is altered in CF nasal epithelial cells, blood cells, and lung macrophages.^41^ However, it is still unclear whether this modification is a trigger or a result of the disease.^42^ CFTR expression is regulated by a housekeeping promoter and distal cisregulatory elements.^43^ Comparison of fetal and adult tissues of lung, colon, and intestine revealed that CFTR expression was reduced during development as a result of DNA methylation. In fetal tissues, CFTR expression is not related to promoter methylation, while in adult individuals the CFTR gene is silenced, suggesting how epigenetic regulation could be correlated to the CF phenotype. In addition, mutant CFTR expression causes the activation of ROS-mediated autophagy. In this kind of cells, the CpG sequence of the CFTR gene results in hypermethylation, causing an alteration of CFTR expression.^34^

Similarly, histone acetylation promotes CFTR expression, but the exact mechanisms of chromatin packaging in the CFTR locus still have to be completely clarified. Histone acetylation neutralizes the positive charge on lysine residues of the amino-terminal tails of the histones, modifying the chromatin packaging and increasing the transcriptional activity.^44^ The basal expression of CFTR gene is connected to an inverted CCAAT element (sequence: 5′-AATTGGAAGCAAAT-3′) located between nucleotides 132 and 119 upstream of the translational start site. CFTR gene hyperacetylation of the CFTR promoter increases gene transcription as a result of major accessibility to the CCAAT sequence.^45^ In human lung and colon, introns 1 and 11 and the promoter result hyperacetylation, suggesting cell-type-specific gene expression of CFTR.^46^

Dysregulation of inflammatory mediators in the CF airway also has an epigenetic basis. A CFTR defect itself causes inflammation, but nevertheless, altered levels of histone acetylation and methylation displayed significant correlations to the increase in inflammatory markers, thus suggesting key epigenetic mechanisms underlying the CF-associated inflammatory phenotype.^47−49^

In conclusion, key epigenetic changes appear to be involved in CF, and they represent promising starting points for further research efforts in this field. In particular, the role of specific epigenetic enzymes, such as the HDAC class of enzymes, deserves particular attention, since multiple pathways associated with CF are closely related to HDAC expression and activity. In this review, special focus will be devoted to HDAC6 isoform because of its unique features and its multifaceted role in CF, which will be discussed in the following paragraphs.

## 3 Structure and Substrates of HDAC6

HDAC enzymes are responsible for acetyl group removal from histones and other protein substrates. Eighteen isoforms of HDAC enzymes have been identified to date, and they are clustered into four classes on the basis of homology to yeast HDACs. Classes I, II, and IV are zinc-dependent HDACs, while class III HDACs are NAD^+^-dependent. HDAC isoforms 1, 2, 3, and 8 (class I) are expressed in the nucleus of the cells of all tissues and share homology with yeast HDAC RDP3.^50,51^ While HDAC isoforms 1 and 2 display nuclear localization, isoforms 3 and 8 can shuttle between the cell nucleus and the cytoplasm. Class II HDACs are subdivided into Class IIA isoforms (4, 5, 7, and 9) and Class IIB isoforms (6 and 10). Class IV contains isoform 11 and shows homology with Class I and II HDACs. Class III HDACs are sirtuins (SIRT isoforms 1–7) and display homology with yeast sirtuin protein Sir2.^50,52,53^

**Figure 4:**
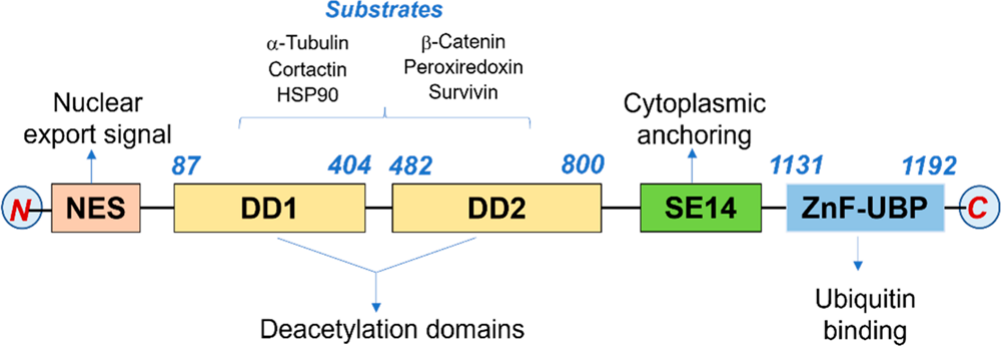
HDAC6 structure: domain organization and substrates.

HDAC6 is a cytoplasmic HDAC isoform containing 1215 amino acids that is encoded by the *hdac6* gene, and the general structure and domain organization are depicted in Figure 4.^54^ The amino-terminal domain is characterized by a nuclear localization signal containing a high percentage of arginine and lysine residues, followed by a nuclear export signal (NES) that is rich in leucine and smooths the export of the enzyme toward its cytoplasmic localization. The center of catalytic activity is represented by two deacetylation domains, called DD1 and DD2, and HDAC6 is the only HDAC enzyme featuring two catalytic domains. The catalytic domains are followed by a serine–glutamic acid (SE14) tetradecapeptide signal that is responsible for cytoplasmic retention (Figure 4). The carboxy-terminal domain is characterized by a ubiquitin binding domain containing Zn^2+^ ion (ZnF-UBP) that is rich in cysteine and histidine residues and can interact with ubiquitinated proteins that undergo the degradation process. In particular, the HDAC6 carboxy-terminal domain controls the transfer of ubiquitinated proteins on the aggresomes present on the microtubules; therefore, HDAC6 binds to ubiquitin, thus preventing protein degradation via the ubiquitin proteasome.

The main HDAC6 substrates are non-histone proteins, namely, heat shock protein 90 (HSP90), α-tubulin, cortactin, and peroxiredoxins (Figure 4).

The first identified HDAC6 substrate was α-tubulin. Reversible acetylation of α-tubulin on residue lysine 40 plays a fundamental role in the regulation of stability and function of microtubules.^55^ Non-acetylated α-tubulin monomers interact with each other through electrostatic interactions, which are lost when the lysine nitrogen is acetylated, thus reducing inter-protofilament interactions and enhancing the flexibility of microtubules under mechanical stress.

Chemotactic cell movement, cell invasion, and migration are triggered by overexpression of HDAC6. Acetylated α-tubulin levels are also responsible for the fine-tuning of motor-based trafficking, which is essential for the transport of cargos along with the microtubule network (Figure 4).^56^ The ratio between acetylated and non-acetylated tubulin is often altered in several pathological conditions and coincides with an overexpression of HDAC6.^57−59^

Another crucial deacetylation substrate is HSP90, an ATP-dependent chaperone involved in the maturation of several proteins, including steroid receptors and mutant p53 proteins. HDAC6 inhibition increases acetylated HSP90 (Ac-HSP90) levels, causing its dissociation from cochaperone p23, which leads to failure of glucocorticoid receptor protein maturation, with important consequences for the transcription mechanism.^60^ Another consequence of the increased Ac-HSP90 levels is the dissociation of the complex between HDAC6 and heat shock transcription factor 1-(HSF1)–HSP90 which in turn triggers the activation of HSF1 and the expression of crucial cellular chaperones.^61^

HDAC6 also performs its deacetylating activity on other two protein substrates, namely, peroxiredoxins I (Prx I) and II (Prx II), both of which are deeply implicated in redox regulation processes.^62^ At low H_2_O_2_ concentrations, peroxiredoxins behave as antioxidants;^63,64^ on the contrary, increased levels of H_2_O_2_ cause the oxidation of their cysteine residue to the corresponding sulfonic acid, prompting the generation of high-molecular-mass protein complexes. Since acetylated Prx is more effective in reducing H_2_O_2_ levels than its non-acetylated counterpart, it is likely that inhibiting HDAC6 function may increase the antioxidant activity of these proteins.

Beyond its interaction with non-histone substrates, HDAC6 interacts with diverse proteins (e.g., ubiquitin, tau, IIp45, and EGFR) through protein–protein interactions, which modulate its deacetylase activity.^14,65^

Owing to its unique functional and structural characteristics, HDAC6 deserves a special role in the armamentarium of epigenetic targets. Accordingly, several research efforts have been devoted to the development of novel, potent, and selective HDAC6 inhibitors for several disease states.^66−70^ An increasing number of reports highlight the potential of selective HDAC6 inhibitors in noncancerous conditions and rare diseases.^14^ In particular, the role of this HDAC isoform, acting as a key player in diverse inflammatory states and a crucial regulator of immune response, is particularly attractive.^71,72^ In this context, recently reported X-ray cocrystal structures of HDAC6 in complex with selective inhibitors will further help refining the design of novel chemical entities with improved potency and selectivity features.^69,73−76^ This Perspective will examine the multifaceted role of HDAC6 in CF and highlight the potential of selective HDAC6 inhibitors as innovative therapeutic options for the treatment of several aspects of this disabling and lethal rare disease.

## 4 The Multifaceted Role of HDAC6 in Cystic Fibrosis

### 4.1 Impaired Microtubule Acetylation in Cystic Fibrosis

**Figure 5:**
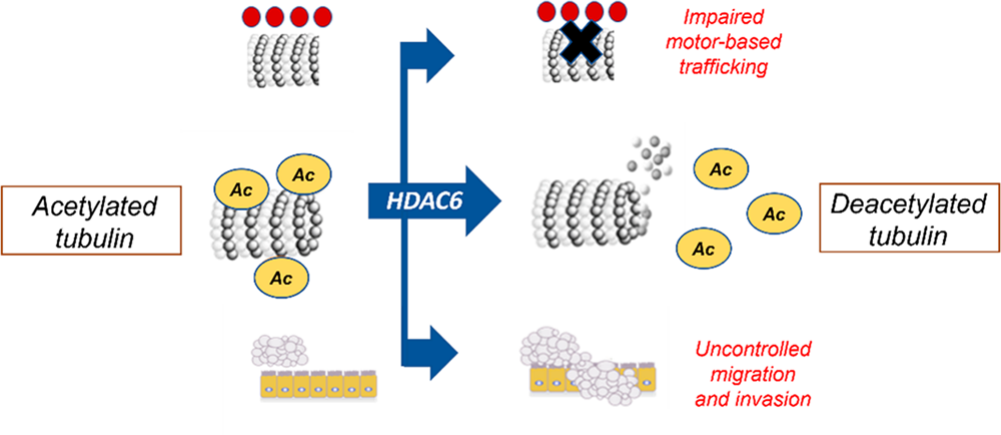
Effects of HDAC6 on α-tubulin, trafficking, and invasion.

As mentioned in the previous paragraphs, defective CFTR is responsible for reduced transepithelial chloride transport, hyperabsorption of sodium, dehydration of epithelial surfaces, and altered inflammatory responses.^77−79^ Interestingly, mutated CFTR has also been related to perinuclear free cholesterol accumulation as a result of defective endosomal transport.^80^ The effectiveness of endosomal transport is a direct consequence of the microtubule network stability and the bidirectional endocytic trafficking (Figure 5).^81,82^ In particular, the microtubule network undergoes extensive post-translational modifications (PTMs), including acetylation, polyglutamylation, polyglycylation, and carboxy terminal cleavage. PTMs are largely responsible for fine-tuning of the microtubule network stability as well as regulation of motor proteins and recruitment of microtubule-associating proteins.^83−86^ Acetylation is a reversible process controlled by histone acetyltransferases, which exert their activity on residue Lys40 of α-tubulin.^87−89^ In contrast, the histone deacetylase enzymes are their counterparts appointed for the hydrolysis of *N*-acetyl groups from pertinent Lys residues. In particular, α-tubulin was the first acknowledged HDAC6 protein substrate. Accordingly, increased expression of HDAC6 promotes tubulin hypoacetylation and chemotactic cell movement (Figure 5).^90−92^ Acetylated α-tubulin also finely controls motor-based trafficking mediated by motor proteins, including kinesin-1 and dynein (Figure 5).^93^ Very recent studies also identified and characterized the disordered N-terminal region of HDAC6 as a microtubule-binding domain, with a microtubule-binding motif spanning two positively charged areas including residues Lys32 to Lys58. The deacetylase and microtubule-binding domains display crucial crosstalk that is critical for the recognition and effective in vitro and in vivo deacetylation of free tubulin dimers, thus revealing the complexity of the recognition process between tubulin and HDAC6 and demonstrating once again that domains external to the tandem catalytic core are crucial for effective substrate deacetylation.^94^ Therefore, perturbation of the HDAC6 function is strongly implicated in the alteration of tubulin function.

This evidence supported the hypothesis that the microtubule acetylation status is responsible for the defective endocytic trafficking and perinuclear cholesterol accumulation detected in CF.^15^ The pivotal function of microtubule regulation in CF was demonstrated in cell and tissue models, and endoplasmic reticulum (ER) stress was recognized as one of the causes of the microtubule alteration in the CF epithelium.^15,17^

In particular, to investigate the mechanisms causing reduced trafficking in the epithelial cells in the case of defective CFTR function, the levels of Ac-tubulin were assessed in IB3 cells (human epithelial cells bearing the ΔF508 mutation) and CFTR-corrected S9 cells by densitometry analysis. As expected, reduced levels were unveiled in IB3 cells, as was perinuclear cholesterol accumulation with respect to WT S9 cells. Reduced Ac-tubulin levels were also confirmed in a primary tissue CF model, namely, the mouse nasal epithelium (MNE), where a 40% decrease in Ac-tubulin level was registered in *Cftr*^*–/–*^ mice relative to WT mice.^15^

Since comparable HDAC6 expression levels were found in IB3 and S9 cells, the decreased Ac-tubulin content in CF cells and tissue was possibly ascribable to defective HDAC6 regulation in the absence of CFTR function. To assess whether HDAC6 inhibition would revert the CF phenotype, both IB3 and S9 cells were subjected to treatment with the selective HDAC6 inhibitor tubastatin A (TubA, **16**; Figure 9 and Table 1) at 10 μM for 24 h. TubA-treated IB3 cells displayed increased levels of Ac-tubulin comparable to those for S9 cells as well as relocation of accumulated perinuclear cholesterol, as detected by monitoring the trafficking of fluorescent 25-[*N*-[(7-nitro-2,1,3-benzoxadiazol-4-yl)methyl]amino]-27-norcholesterol (NBD-cholesterol). A further confirmation that the effects of TubA were only related to selective HDAC6 inhibition was provided by knockdown of HDAC6 expression in both IB3 and S9 cells using shRNA against HDAC6. In support of the clear role of HDAC6, the silenced IB3 cells displayed increased Ac-tubulin levels, although the effects of acute HDAC6 inhibition through pharmacological treatment were more marked than the effects of gene silencing (3-fold vs 1.5-fold increase in Ac-tubulin content, respectively).^15^

As mentioned before, chronic ER stress has been related to decreased Ac-tubulin content in CF. ER stress in CF cells has been associated with misfolding of ΔF508 CFTR and also represents a trigger for aggresome formation.^11,95−97^ Accordingly, it has been shown that thapsigargin, a molecule leading to ER stress and activation of the unfolded protein response,^98^ is able to reduce Ac-tubulin levels and recapitulate the cholesterol accumulation phenotype in S9 cells.^15^ The same study also showed that IB3 cells and MNE from *Cftr*^*–/–*^ mice display reduced Ac-tubulin levels and higher levels of GRP78, an inherent marker of ER stress.^99^ To further validate the role of ER stress associated with the CF phenotype, it was demonstrated that ΔF508 CFTR corrector C18^100^ (10 μM for 72 h) was able to increase the Ac-tubulin content to a comparable extent with respect to TubA; however, the effect on cholesterol mobilization was inferior to that exerted by TubA treatment.^15^ As a further effort to investigate more specific molecular mechanisms linking ER stress and tubulin acetylation status, the role of phosphatidylinositol 3-kinase p110α (PIK3CA) was examined because of its direct ability to associate with HDAC6 and respond to diverse cellular stresses.^101,102^ Accordingly, the use of the PIK3CA inhibitor PIK-75 (0.5 μM for 24 h) evoked a relevant increase of Ac-tubulin levels in IB3 cells and led the cholesterol distribution to a more WT phenotype.^15^

Interestingly, the use of 4-phenylbutyrate (4-PB, **17**; Figure 9 and Table 1) as an ER stress reliever at 1 mM for 48 h^103,104^ resulted in a significant increase in Ac-tubulin levels and reduced perinuclear accumulation of cholesterol in IB3 cells. Since 4-PB also acts as a pan-HDAC inhibitor,^105,106^ the role of an HDAC6-mediated mechanism for the observed effects cannot be ruled out.

A few years later, more direct evidence was provided that HDAC6 activity influences the levels of membrane cholesterol in CF epithelium and that the electrochemical measurement of these levels is closely linked to genetic and pharmacological CFTR correction.^107^ In particular, the evidence demonstrating that high cholesterol levels in CF cells are correlated with the CFTR phenotype and depend on de novo cholesterol synthesis^108−110^ prompted the hypothesis of an adaptative response to the loss of CTFR function. On the basis of this assumption, it is reasonable to presume that electrochemical determination of membrane cholesterol levels can serve as a biomarker to observe the adjustment of intracellular events upon administration of novel therapeutics in CF.

First, the expression of HDAC6 was knocked out from a ΔF508 mouse, and then the membrane cholesterol amount was electrochemically evaluated by the use of an electrode with cholesterol oxidase. The study demonstrated that HDAC6 depletion causes an almost 2-fold increase in membrane cholesterol levels, thus restoring correct cholesterol processing. The effects of pharmacological inhibition of HDAC6 using TubA (10 μM for 24 h) on membrane cholesterol levels were also assessed in CF bronchial epithelial cells (CFBE) through a double-pulse technique.^107^ These experiments conclusively demonstrated that tubulin acetylation is a crucial pathway in pathophysiology of several CF phenotypes and that HDAC6 inhibition holds promising potential as innovative therapeutic option for restoring those pathways to a more WT-like pattern.

As a further support to this evidence, it was very recently demonstrated that resveratrol (RSV, **18**; Figure 9 and Table 1) is able to restore intracellular transport in CF epithelial cells.^111^ RSV is well-characterized as an activator of SIRT1,^112^ although it also acts as a pan-HDAC inhibitor and antagonist of peroxisome proliferator-activated receptors PPARγ and PPARα.^113,114^ RSV treatment (50 μM, 24 h) in IB3 cells and control S9 cells effectively reversed the perinuclear cholesterol accumulation, suggesting that intracellular transport is restored.^111^

To assess whether a correctly functioning CFTR was essential for the intracellular transport correction in CF cells, RSV was tested in cells isolated from *Cftr*^–/–^ mice to check the improvement of cholesterol mobilization in MNE. RSV (50 μM for 24 h) significantly lowered cholesterol accumulation in *Cftr*^–/–^ MNE cells, demonstrating that CFTR function is not critical for RSV efficacy. The study also showed that RSV at the same concentration is able to trigger microtubule formation in both IB3 cells and human nasal epithelial (HNE) cells from ΔF508 patients and that these effects are not mediated by sirtuin signaling, as demonstrated by cotreatment with the SIRT1 inhibitor EX-527 (IB3 cells, 1 μM for 24 h), which did not have a significant impact on the efficacy of RSV. It was then hypothesized that the influence of RSV on microtubules could be ascribable to PPARγ activation and HDAC6 inhibition. Accordingly, cotreatment with the PPARγ inhibitor GW-9662 (IB3 cells, 20 μM, 24 h) demonstrated that the effects of RSV in promoting intracellular transport are partially mediated by PPARγ receptors. Conclusive support for the role of HDAC6 inhibition on the effects of RSV came from the evidence that treatment with RSV (50 μM, 24 h) markedly enhances tubulin acetylation in IB3 cells.^111^

Although additional studies are needed, the observation that RSV reverses CF cellular phenotypes more effectively than ibuprofen^115^ suggests that RSV could represent a valuable anti-inflammatory treatment with a multifaceted profile that is in part ascribable to its ability to inhibit HDAC6. However, other reports demonstrate that RSV is able to prevent the accumulation of acetylated tubulin resulting from mitochondrial damage, precluding inflammasome activation.^116^ It should also be mentioned that the main limitation of RSV is its low water solubility and the impossibility of reaching plasma levels even remotely close to the concentration of 50 μM utilized in this and other cellular studies.^117^

Taken together, these data validate the role of HDAC6 as a key player in microtubule acetylation status and trafficking in CF. TubA, behaving as a selective HDAC6 inhibitor, and RSV, through pan-HDAC inhibition and PPARγ activation, served as chemical probes for a deeper understanding of CF cell signaling related to tubulin acetylation status, thus providing a glimpse of a new therapeutic avenue for effective reversal of CF phenotype.

### 4.2 HDAC6 and Bacterial Clearance in Cystic Fibrosis

**Figure 6:**
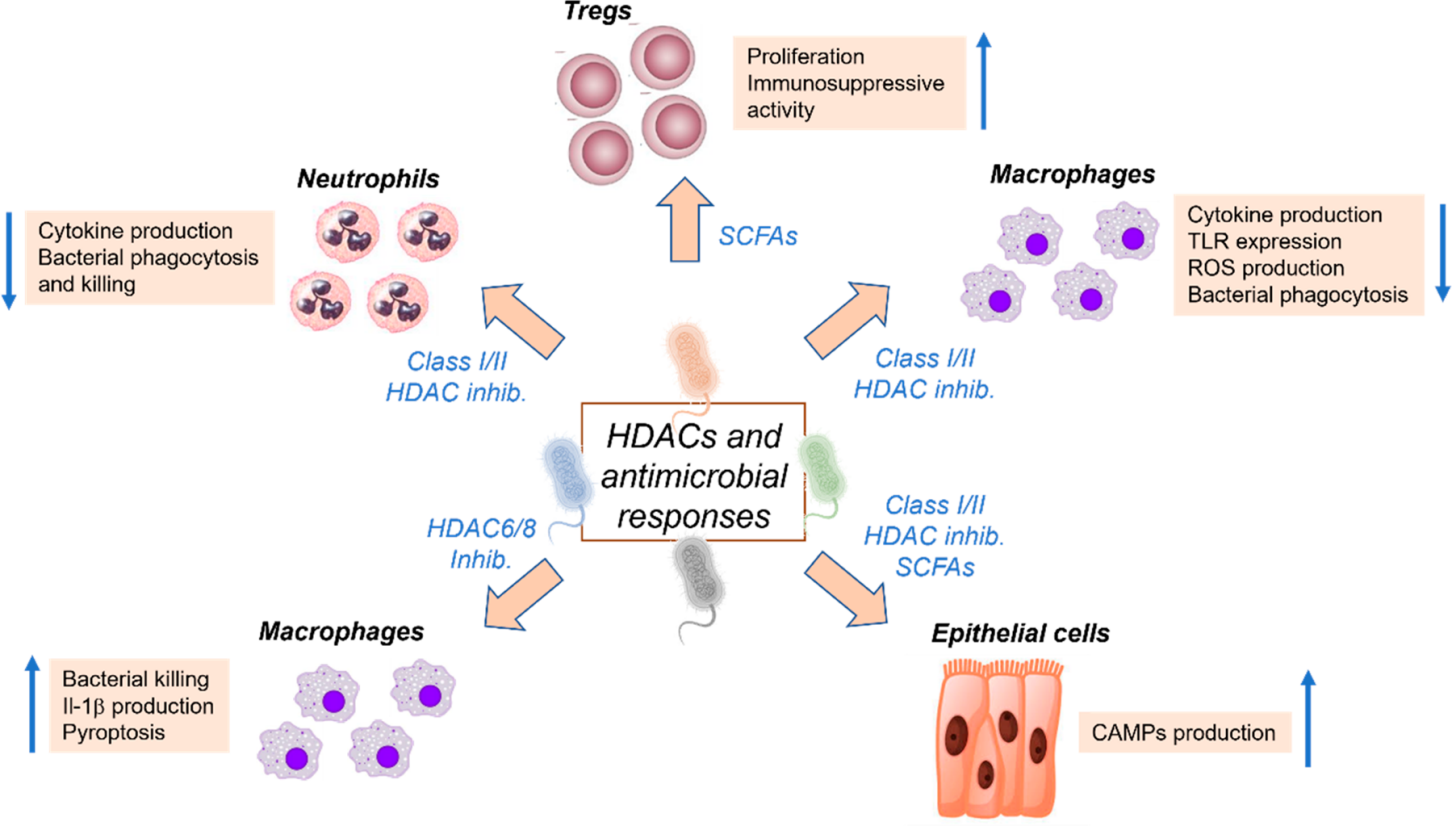
Regulation of antimicrobial responses by HDAC inhibitors.

Among protein PTMs, reversible acetylation deserves a primary role among the processes involved in the responses to environmental stimuli as well as cellular homeostasis. In particular, the evidence supporting dynamic regulation of epigenetic marks by environmental cues has prompted research efforts toward the elucidation of epigenetic pathways in microbial infections.^118^ Accordingly, huge progress has been made lately on the bacterial regulation of the host acetylation system, with a special focus on the mechanisms engaged to evade immune response.^119,120^ In general, pathogenic microorganisms utilize a limited number of mechanisms involving acetylation of histone and non-histone proteins, including modulation of the activity and expression of HAT and HDAC enzymes thorough diverse bacterial effectors as well as the production of metabolites regulating the so-called “acetylome”.^118^ In this context, isoform HDAC1 may be considered the crucial acetylation system targeted by pathogens for evading the host immune system, as demonstrated by the efficacy of silencing of HDAC1 expression or enzymatic inhibition in restoring defense gene expression.^121^ More recently, it was demonstrated that the quorum-sensing signal generated by *PA*, namely, 2-aminoacetophenone, prompted the expression of HDAC1 in human THP-1 monocytes, thus causing hypoacetylation of histone H3K18; this in turn led to reduced induction of inflammatory cytokines and chemokines, such as TNF, IL-β, and MCP-1. The process was also demonstrated to be completely reverted by HDAC1 inhibition, thus unambiguously highlighting the role of this isoform in promoting tolerance to *PA* (Figure 6).^122^

As mentioned above, metabolites produced by pathogenic bacteria can also modulate the host acetylation system. In particular, it has been demonstrated that short-chain fatty acids (SCFAs) such as propionic acid (**19**, Figure 9 and Table 1) and butyric acid (**20**, Figure 9 and Table 1) produced by anaerobic bacteria behave as inhibitors of class I/II HDACs, thus regulating several facets of immune response (Figure 6).^123^ Accordingly, treatment with butyrate prompted histone H3 acetylation at the promoter region of the transcription factor FoxP3, which is crucial in the differentiation of regulatory T cells. In this context, isoforms HDAC6 and HDAC9 demonstrated a suppressive role in FoxP3 induction (Figure 6).^124^

Around a decade ago, pioneering studies started unveiling the effect of HDAC inhibitors on microbial infection. In particular, it was demonstrated that the use of valproic acid (VPA, **21**; Figure 9 and Table 1) increased mortality in mice upon nonsevere infection by *Klebsiella pneumoniae* or *Candida albicans*(125) and that HDAC inhibition was able to reduce phagocytosis and killing of *E. coli* and *SA* by bone-marrow-derived macrophages.^126^

These initial observations supporting an unfavorable outcome when HDAC inhibitors are used during bacterial infection were controverted by subsequent studies. Indeed, it was clarified that the effects of HDAC inhibitors on antibacterial response strongly relied upon the isoform selectivity and timing of the treatment. Interestingly, in 2015 Ariffin et al. highlighted that only the HDAC6-selective inhibitor TubA and not MS-275 (entinostat, **22**; Figure 9 and Table 1), which specifically targets class I HDACs, enhanced bacterial killing by macrophages (Figure 6).^127^ The same study also highlighted that acute treatment with HDAC inhibitors at the insurgence of bacterial infection boosts mitochondrial ROS production by human macrophages, which is in line with an increased antibacterial response. Almost parallel studies also showed that HDAC6-selective inhibition through the use of TubA promoted bacterial clearance, reduced pro-inflammatory cytokine production, restored innate immune cell populations in the bone marrow, and improved survival in a mouse model of sepsis (Figure 6).^128,129^ These data, together with the evidence linking HDAC6 to control of mitochondrial function and stimulation of mitochondrial ROS production, suggest a crucial role for HDAC6 in regulating bacterial clearance,^130−132^ while class I HDACs seem unlikely to exert such an effect.

While the previously described reports mainly focus on the effects of HDAC inhibitors on immune cell function, several experiments analyzing the responses of epithelial cells to bacterial challenges support the role of HDAC inhibitors as effective regulators of the production of cationic antimicrobial peptides (CAMPs) (Figure 6).^133^ In particular, the expression of defensins and cathelicidins, the two main classes of mammalian CAMPs, is robustly triggered by HDAC inhibitors in colonic and airway epithelial cells.^134−138^ Although induction of CAMPs by several HDAC inhibitors has been widely documented across several types of epithelial cells, the mechanisms underlying this effect are mostly unclear or at the stage of preliminary investigation.

Collectively, these findings underline the dual-faceted potential of HDAC6 inhibitors as anti-infective agents through either promotion of innate immune-mediated bacterial clearance or reduction of the damage induced by excessive inflammation. These data also highlight the potential to use HDAC6-selective inhibitors for treatment of chronic inflammatory diseases of the airways.

To further validate these premises, a recent study examined the outcome of *Hdac6* depletion on both the CF inflammatory response and the bacterial load in a model of infection using clinical *PA* isolates embedded in agarose beads, which effectively recapitulates CF phenotype.^18^ In that study, genetic ablation was preferred over pharmacological inhibition in order to avoid contributions ascribable to off-target effects. The loss of *Hdac6* was demonstrated to increase the rate of bacterial clearance in CF mice, thus restoring CF responses to bacterial challenge. These data, coupled with the limited weight loss and the regulation of neutrophil recruitment upon *Hdac6* depletion demonstrated in the same study, further depict HDAC6 as a key regulator in several secondary phenotypes associated with impaired CFTR function and support the potential benefits of using HDAC6-selective inhibitors in CF patients independently of CFTR phenotype.

### 4.3 HDAC6 in the Regulation of Inflammatory and Fibrotic Phenotypes in Cystic Fibrosis

The complex CF phenotype is associated with several clinical manifestations, although lung disease, chronic airway inflammation, and infection are by far the main causes of morbimortality. A common thread of the end stage of CF-associated lung disease is represented by extensive pulmonary fibrosis, characterized by increased collagen deposition and tissue remodeling.

Fibrosis is generated by overgrowth of various tissues and an increased amount of myofibroblasts and it is flanked by anomalous deposition of extracellular matrix components, a process known as epithelial–mesenchymal transition. EMT is a crucial process in CF involving loss of cell–cell junctions and polarization of cell-surface molecules of epithelial cells, which thus acquire the characteristics of mesenchymal cells.^5,139^ Beyond their role as a key scaffold for parenchymal tissue, fibroblasts can secrete key inflammatory chemoattractants, such as chemokine C–C ligand-2 (CCL-2) and CCL-8, interleukin-16 (IL-16) and IL-8, regulated on activation normal T-cell expressed and secreted (RANTES), and monocyte chemotactic protein-1 and -2.^140,141^ The dysregulated fibroblast phenotype in CF was investigated in a bleomycin-induced fibrosis model in ΔF508 homozygous mice.^5^ An increased release of TGF-β1, a potent EMT inducer, was demonstrated in CF lungs. Members of the TGF-β family are potent inducers of EMT, and overexpression of TGF-β1 was also previously linked with a more severe CF lung phenotype.^142^ Moreover, CF lungs challenged with bleomycin were also shown to overexpress TIMP-1, a predictive marker of tissue remodeling reflecting a protease imbalance in damaged lungs.^5,143^ The study also demonstrated that upon inflammatory stimulation, mRNA and protein expression levels of proinflammatory mediators (CCL-2, TNF-α, IL-16 and IL-18) were higher in CF than in wild-type fibroblasts, thus demonstrating that dysregulated proinflammatory and fibrotic status in CF fibroblasts is an extensive and multifaceted process involving several signaling pathways and transcription factors.^5^

The demonstration of the close crosstalk between inflammation and fibrosis in CF opened up possibilities for novel disease-modifying treatments specifically targeting these mechanisms.

#### 4.3.1 HDAC6 and Inflammatory Responses in Cystic Fibrosis

**Figure 7:**
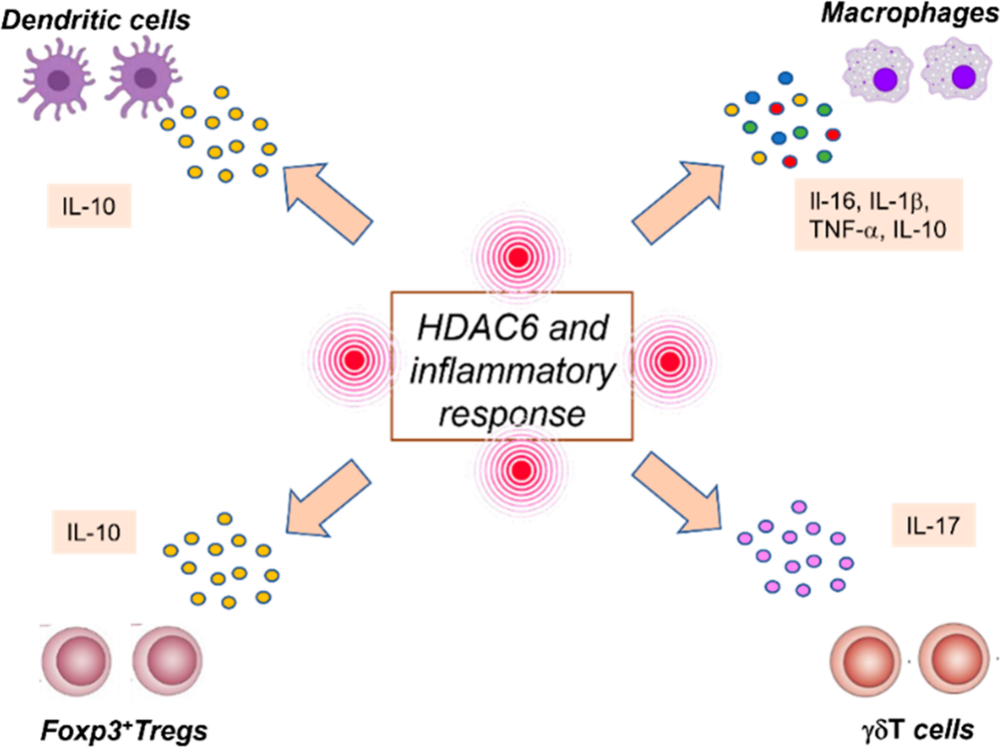
Role of HDAC6 in the regulation of inflammatory cells (macrophages, dendritic cells, γδT cells, FoxP3^+^ regulatory T cells) and cytokines (IL-6, IL-1β, TNF-α, IL-10, and IL-17).

In the context of inflammation, increasing evidence is available highlighting the key role of HDACs and HDAC inhibitors in the regulation of chemokines and cytokines (Figure 7).^144^ HDAC inhibitors, in particular, have been shown to downregulate several pathways involving cytokines, chemokines, and growth factors, thus controlling key inflammatory pathways in inflammatory diseases with different origins.^145,146^ With respect to cytokines, the pan-HDAC inhibitors trichostatin A (TSA, **23**; Figure 9 and Table 1) and romidepsin (**24**, Figure 9 and Table 1) were found to suppress IFN-α-induced transcriptional responses, thus regulating both innate and adaptative immunity,^147,148^ while the pan-HDAC inhibitors SAHA (vorinostat, **25**; Figure 10 and Table 1) and TSA were demonstrated to decrease the expression of IFN-γ in various disease models.^149^ Interestingly, a predominant role of isoform HDAC6 in regulation of cytokines has been unveiled by several studies. Accordingly, the HDAC3/6/8 inhibitor MC2625 (**26**, Figure 10 and Table 1) and the HDAC6-selective inhibitor MC2780 (**27**, Figure 10 and Table 1) were shown to downregulate IL-1β expression in epithelial, fibroblast, and myogenic cell lines,^150^ and HDAC6 was identified as a key player in the regulation of NF-κB activity, which is related to IL-1β signaling.^72^ Selective HDAC6 inhibition by TubA was also linked to inhibition of IL-6 in an arthritis mouse model.^151^ However, the effects of HDAC6 inhibition on the production of the anti-inflammatory cytokine IL-10 are still controversial.^150,152^

The involvement in HDACs in chemokine signaling was also supported by several experiments. In particular, it was demonstrated that pan-HDAC inhibition induced the expression of CCL-2^153^ and that both HDAC6-selective inhibition with CKD506 (**28**, Figure 10 and Table 1) and pan-HDAC inhibition were able to decrease CCL-4 expression.^154,155^

HDAC6 has also been shown to play a critical role in the regulation of inflammatory cells. For example, inhibition of HDAC6 functions causes stimulation of inflammatory antigen-presenting cells with a key role in the induction of T-cell activation and T-cell tolerance.^72,156,157^ Moreover, depletion of HDAC6 has been demonstrated to endorse the suppressive activity of FoxP3^+^ regulatory T-cells in inflammation models and to increase the population of γδT cells, which are involved in the release of IL-17.^158^

According to these data, targeted HDAC6 inhibition has been increasingly proposed over the last year as a promising therapeutic strategy toward inflammatory disorders, including airway inflammation.^71^ For example, TubA has been demonstrated to efficiently relieve airway inflammatory state and hyper-responsiveness in a chronic mouse model of allergy, thus opening up the possibility for a novel therapeutic option in asthma treatment.^159^ Also, TubA significantly inhibited cigarette-smoke-induced airway dysfunction, thus paving the way to an innovative therapeutic strategy against chronic obstructive pulmonary disease (COPD).^160^

With respect to CF, the potential usefulness of HDAC inhibitors in correcting the ΔF508 CFTR variants was recently evaluated in several studies.^16,161,162^ In particular, the pan-HDAC inhibitors panobinostat (**29**, Figure 10 and Table 1) and romidepsin provided functional correction of Class II and III CFTR variants, as they were able to cause dose-dependent enhancement of the expression of ΔF508 CFTR and restore the activity of the cell-surface chloride channel in primary human bronchial epithelial cells.^161^ Most importantly, the effects of the combination of panobinostat and romidepsin with lumacaftor were evaluated. Panobinostat (2.5 nM) and romidepsin (0.6 nM) were combined with a higher dose of VX809 (3 μM). While the combination with romidepsin produced an additive effect on the trafficking efficiency of ΔF508 CFTR, the combination with panobinostat unveiled a synergism in improving the trafficking efficiency after immunoblotting and quantification analysis of CFTR expression in human CFBE-ΔF508 cells.^161^ A more inflammation-oriented study evaluated the effect of pan-HDAC inhibition in *PA* lipopolysaccharide (*PA*-LPS)-induced airway inflammation and CF lung disease.^16^ HDAC inhibition through SAHA was shown to control TNF-α-induced IL-8 and NF-κB reporter activity, suggesting that HDAC inhibitors regulate TNF-α-induced inflammation and CF lung disease. CFTR^–/–^ mice treated with *PA*-LPS (20 μg/mouse) were used as a model for CF-induced lung infection. Intratracheal administration of SAHA (100 μg/mouse daily for 3 days) demonstrated that SAHA was able to efficiently control *PA*-LPS-induced IL-6 levels in mice treated for 1 day, while treatment for 2 or 3 days was shown to be detrimental. The outcome of a lower dose (50 μg/mouse) was then assessed, demonstrating that *PA*-LPS-induced IL-6 and myeloperoxidase levels were efficiently reduced by SAHA and highlighting its role in controlling chronic airway inflammation and neutrophil activity. Moreover, the same study demonstrated that NF-κB activation (as a marker of inflammation) and Nrf2 regulation (as a marker of antioxidant response) are effectively corrected by SAHA treatment.^16^ In agreement with previous studies, SAHA demonstrated the ability to restore trafficking in misfolded ΔF508 CFTR and to promote FoxP3^+^ T-reg activity, thus providing conclusive evidence of the role of HDAC inhibition in modulation of innate and adaptive immune response linked to pathogenesis and progression in CF-related inflammatory lung disease.^16^

More recent evidence pointed out the prevalent role of HDAC6 in the beneficial effect of HDAC inhibition especially with respect to the inflammatory phenotype in CF, thus supporting the evaluation of HDAC6-selective inhibitors in this context. Accordingly, it was recently demonstrated that HDAC6 depletion was able to improve CF mouse airway inflammatory responses to bacterial challenge.^18^ A clinical *PA* isolated by means of the agar bead model of infection was employed. This model nicely recapitulates the CF phenotype related to an excessive inflammatory response, with particular reference to the increased neutrophil recruitment.

The loss of *Hdac6* on a CF background increased the rate of bacterial clearance, and this effect was ascribed to a crucial regulatory step in CF immune responses. Accordingly, it was shown that HDAC6 controls neutrophil recruitment in CF mice.^18^ Reduced cytokine production in *Hdac6*-depleted CF mice was also demonstrated in the same study.

#### 4.3.2 HDAC6 and Fibrotic Remodeling in Cystic Fibrosis

Aberrant HDAC activity and/or expression is detected in fibrotic diseases, and increasing amounts of data support the role of HDACs in the initiation and evolution of fibrotic phenotype in several organs, including lungs, heart, liver, and kidneys.^163^

**Figure 8:**
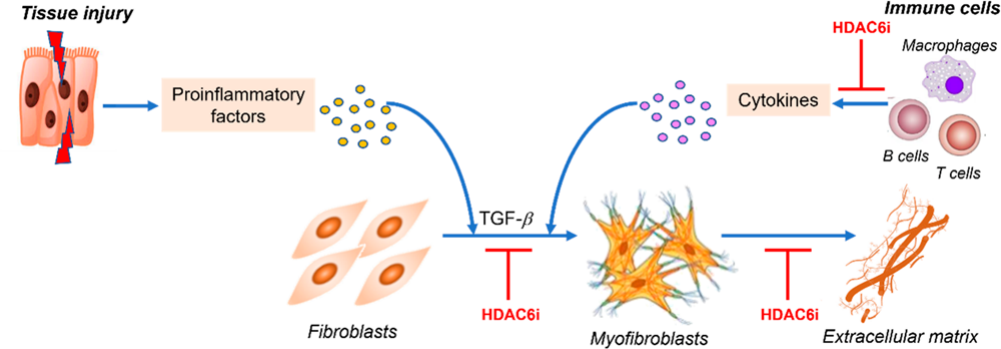
Role of HDAC6 in regulation of the fibrotic process. Injured tissues or activated immune cells induce the production of profibrotic factors, in turn prompting fibroblast differentiation into myofibroblasts, which actively synthesize extracellular matrix. HDAC6 inhibitors negatively regulate the fibrotic process by acting at different levels of the profibrotic cascade.

More specifically, the regulation of TGF-β1 by HDAC6 is crucial in the initiation and progression of fibrotic diseases through EMT (Figure 8).^14,164,165^ Recent data provided evidence that HDAC6 blockade by siRNA or tubacin (**30**, Figure 10 and Table 1) is able to reduce TGFβ1-induced EMT markers and SMAD3 activation in response to TGF-β1. Since SMAD3 is a key player in TGFβ1 signaling, its deactivation impairs HDAC6-dependent deacetylation of α-tubulin, highlighting the crucial importance of HDAC6 in EMT through the TGF-β1–SMAD3 signaling pathway.^19^ Several lines of evidence demonstrated the effectiveness of pan-HDAC inhibitors (including romidepsin, SAHA, and panobinostat) against idiopathic lung fibrosis (IPF) and fibrotic diseases, mainly based on the decrease of myofibroblast differentiation and fibroblast proliferation prompted by TGF-β1.^166−168^ More recent reports pointed out the efficacy of a targeted HDAC6 approach in IPF. Accordingly, TubA was shown to protect mice from lung fibrosis by repressing TGF-β1-induced collagen expression and diminished Akt phosphorylation,^169^ and novel indoline-based inhibitors demonstrated efficacy in a human lung model of TGF-β1-dependent fibrogenesis and the ability to inhibit fibrotic sphere formation.^73^

**Table 1:** IC_50_ Values for HDAC Inhibitors **16**–**30** on HDAC6 and Other Isoforms

	IC_50_ (μM)	
compound	HDAC6	other HDACs
tubastatin A (**16**)^170^	0.015	HDAC1, 16.4
		HDAC2, >30
		HDAC3, >30
		HDAC8, 0.85
sodium 4-phenylbutyrate (**17**)^171^		HDAC1, 162
resveratrol (**18**)^114^	32% (100 μM)^a^	HDAC1, 39% (100 μM)^a^
		HDAC2, 21% (100 μM)
		HDAC4, 29% (100 μM)
		HDAC4, 50% (100 μM)
		HDAC4, 23% (100 μM)
propionic acid (**19**)	–	–
butyric acid (**20**)^172^	–	HDAC1, 300
		HDAC2, 400
		HDAC7, 300
valproic acid (**21**)^171^	–	HDAC1, 40
entinostat (**22**)^173^	>100	HDAC1, 0.18
		HDAC3, 0.74
trichostatin A (**23**)^174^	0.009	HDAC1, 0.006
romidepsin (**24**)^175^	0.79	HDAC1, 0.0016
		HDAC2, 0.0039
SAHA (**25**)^176^	0.033	HDAC1, 0.033
		HDAC2, 0.096
		HDAC3, 0.020
		HDAC8, 0.540
MC2625 (**26**)^150^	0.01	HDAC1, 1.42
		HDAC2, 1.77
		HDAC3, 0.080
		HDAC8, 0.61
MC2780 (**27**)^150^	0.011	HDAC1, 2.9
		HDAC2, 2.1
		HDAC3, 10.8
		HDAC8, 1.2
CDK506 (**28**)^68^	0.041	HDAC1, 11.4
panobinostat (**29**)^177^	0.015	HDAC1, 0.015
		HDAC3, 0.015
		HDAC8, 0.55
tubacin (**30**)^92^	0.004	HDAC1, 1.40
		HDAC2, 6.27
		HDAC3, 1.27
		HDAC8, 1.27

a Overall inhibition of human HDAC enzymes in HeLa nuclear extracts.

**Figure 9:**
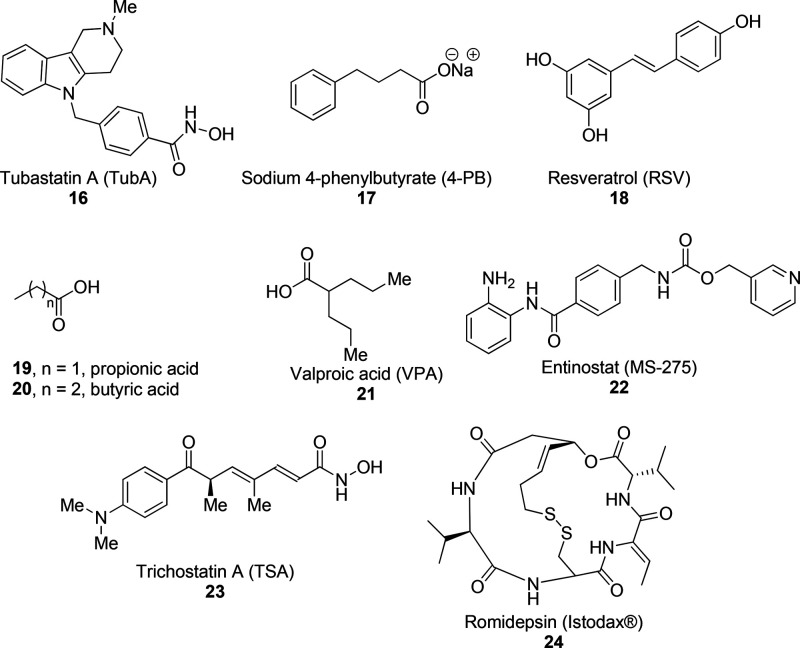
Structures of HDAC inhibitors **16**–**24**.

**Figure 10:**
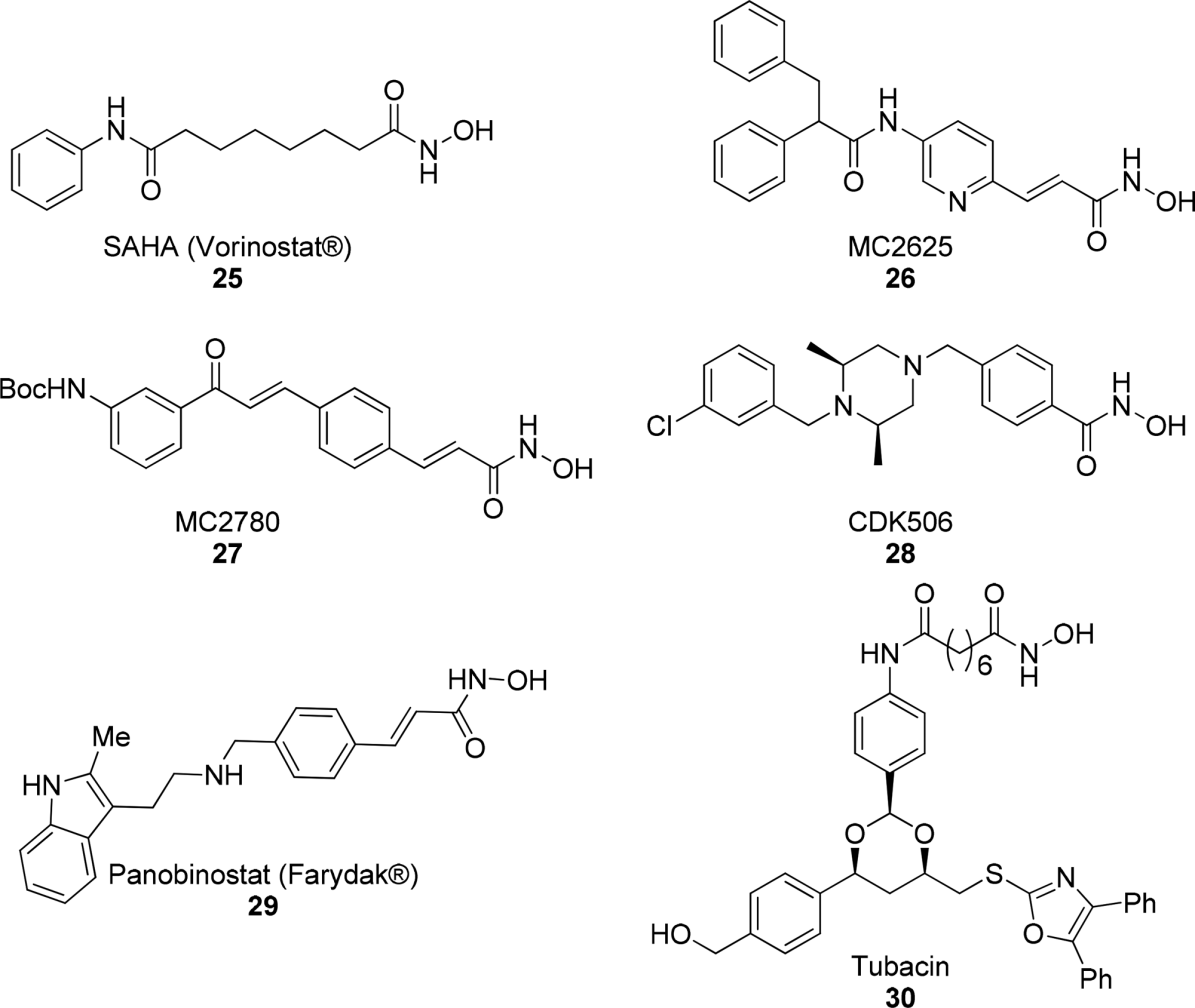
Structures of HDAC inhibitors **25**–**30**.

These data foster further investigation of HDAC6-selective inhibitors as potential therapeutic tools to tackle the fibrotic process in CF and other diseases.^59,163,166,169^

## 5 Perspectives

Increasing evidence points to unveiling a new guise for epigenetic targets as key players in many nononcological diseases, particularly rare diseases. In the context of CF, the past decade has represented a phase of hope for patients, with increased scientific awareness toward novel CF therapeutics. Accordingly, researchers in academia and pharmaceutical companies are devoting intense research efforts to leverage new technologies and scientific knowledge to successfully develop new effective therapies that are changing the patient quality and expectation of life, although there is still the need for great improvement and simplification of the therapy.

The void of knowledge on how the loss of CTFR function is linked to many of the secondary manifestations of CF remains a crucial gap to be filled. Also, there is still a lack of understanding of the molecular underpinnings and crosstalk mechanisms related to inflammatory response, immune system engagement, and microbial infections in CF.

The objective of developing a new inflammatory therapy for CF is closely linked to the necessity of identifying a novel therapeutic option that could allow the resolution of the prolonged and aggressive inflammatory phenotype associated with CF but also guarantee sustained immune response for the eradication of infections.

During the past decade, clear evidence has been provided that the protein acetylation status of the host is dynamically regulated during infection and helps establish an efficient response against pathogens. Recent evidence has also pointed out the role of microtubule acetylation as a crucial regulator of intracellular transport in CF cells and CF-related inflammatory responses.

In this frame, HDAC enzymes appear as key players because of their role in modulating the acetylation balance in several pathophysiological conditions. HDAC6, in particular, displays unique features owing to its distinctive structure, localization and nature of substrates. The concept that several CF phenotypes can be corrected by HDAC6 inhibition is of pivotal importance for those patient populations bearing CFTR mutations not approachable with currently available potentiators and/or correctors. Moreover, HDAC6 inhibitors could also flank CFTR-targeted therapies in order to improve the management of inflammation, fibrosis and infection in CF.

The effects of HDAC6 partially selective modulators and pan-HDAC inhibitors have been recently examined in several cell and animal models of rare diseases, often providing substantial proof-of-concept for their utility as innovative therapeutic option. However, none of the clinically approved HDAC displays selectivity for HDAC6. This means that they cannot work as effective chemical probes to dissect the role of HDAC6 in rare diseases. Moreover, they cannot be considered ready-to-use therapeutics for treating these patients especially due to their therapeutic index, which would not be compatible with a chronic drug administration.

In this context, the increasingly appearing X-ray cocrystal structures of HDAC6 enzyme in complex with several HDAC6 inhibitors are allowing more effective structure-based design strategies, which will certainly help medicinal chemists to drive the drug discovery trajectory toward potent, selective and safer HDAC6 inhibitors, to be employed as novel therapeutic option in several disease states.

## Abbreviation Used

4-PB: 4-phenylbutyrate
Ac-HSP90: acetylated HSP90
ALI: acute lung injury
BALF: broncho-alveolar lavage fluid
BDNF: brain-derived neurotrophic factor
BDNG-YFP: BDNF tagged with a yellow fluorescent protein
CAMPs: cationic antimicrobial peptides
CCL: chemokine C–C ligand
CF: cystic fibrosis
CFBE: CF bronchial epithelial cells
CFTR: cystic fibrosis transmembrane conductance regulator
AMP: adenosine monophosphate
CMT: Charcot–Marie–Tooth disease
CNS: central nervous system
COPD: chronic obstructive pulmonary disease
COX: cyclooxygenase
DD: deacetylation domain
dHMN2B: distal hereditary motor neuropathy 2B
DNMT: DNA methyltransferase
DRG: dorsal root ganglion
*E. coli*: *Escherichia coli*
EGFR: epidermal growth factor receptor
EMT: epithelial–mesenchymal transition
ER: endoplasmic reticulum
FDA: U.S. Food and Drug Administration
GlyRS: Glycyl tRNA synthetase
GR: glucocorticoid receptor
GRP78: 78 kDa glucose-regulated protein
HAT: histone acetyltransferase
HD: Huntington’s disease
HDAC: histone deacetylase
HMN: hereditary motor neuropathies
HMT: histone methyltransferase
HNE: human nasal epithelial
HSF1: heat shock transcription factor 1
HSP90: heat shock protein 90
HSPB1: heat-shock protein B1
IB3: human epithelial cells bearing the ΔF508 mutation
IFN: interferon
IL: interleukin
ILD: interstitial lung disease
IPF: idiopathic pulmonary fibrosis
iPSCs: pluripotent stem cells
IRD: inherited retinal disorder
JIP1: JNK-interacting protein 1
KAT: lysine acetyltransferase
KC: keratinocyte-derived chemokine
LOX: lysyl oxidase
LPS: lipopolysaccharide
LTB4: leukotriene B4
Lys: lysine
MCP-1: monocyte chemoattractant protein-1
MeCP2: X-linked methyl-CpG-binding protein 2
Mip-2: microphage inflammatory protein-2
miRNA: microRNA
MNE: mouse nasal epithelium
MRSA: methicillin-resistant *Staphylococcus aureus*
NBD-cholesterol: 25-[*N*-[(7-nitro-2,1,3-benzoxadiazol-4-yl)methyl]amino]-27-norcholesterol
ncRNA: noncoding RNA
NES: nuclear export signal
NFκB: nuclear factor kappa light-chain enhancer of activated B cells
Nrf2: nuclear factor erythroid 2-related factor 2
NSAID: nonsteroidal anti-inflammatory drug
NTG: nontransgenic mice
NTM: nontuberculous mycobacteria
p97/VCP: p97/valosin-containing protein
*PA*: *Pseudomonas aeruginosa*
PIK-75: PIK3CA inhibitor
PIK3CA: phosphatidylinositol 3-kinase p110α
PLAP: phospholipase A2 inactivating protein
PPAR: peroxisome proliferator-activated receptor
Prx: peroxiredoxin I
PTM: post-translational modification
RANTES: regulated on activation normal T-cell expressed and secreted
ROS: reactive oxygen species
RP: retinitis pigmentosa
RTT: Rett syndrome
RSV: resveratrol
*SA*: *Staphylococcus aureus*
SAHA: suberoylanilide hydroxamic acid
SCFA: short chain fatty acid
SE: serine–glutamic acid
shRNA: shot hairpin RNA
SIRT: sirtuin
SMAD3: small mothers against decapentaplegic family member 3
TGF-β: transforming growth factor-β
TIMP-1: tissue inhibitor of metalloproteinase-1
TSA: trichostatin A
Tub A: tubastatin A
UBP: ubiquitin-specific protease
VEGF: vascular endothelial growth factor
VIPN: vincristine-induced peripheral neuropathies
VPA: valproic acid
WT: wild type
ZnF-UBP: ubiquitin C-terminus hydrolase-like zinc finger
